# Discovery of Oxime Ethers as Hepatitis B Virus (HBV) Inhibitors by Docking, Screening and In Vitro Investigation

**DOI:** 10.3390/molecules23030637

**Published:** 2018-03-12

**Authors:** Jie Tan, Min Zhou, Xinhua Cui, Zhuocai Wei, Wanxing Wei

**Affiliations:** 1 College of Chemistry and Chemical Engineering, Guangxi University, Nanning 53004, China; yulitanjie@163.com (J.T.); eelmm@gxu.edu.cn (M.Z.); cuixinhua123@163.com (X.C.); liveupto@sina.cn (Z.W.); 2 Guangxi Colleges and Universities Key Laboratory of Applied Chemistry Technology and Resource Development, Guangxi University, Nanning 53004, China

**Keywords:** synthesis, oxime ethers derivatives, anti-HBV activity, molecular docking

## Abstract

A series of oxime ethers with C_6_-C_4_ fragment was designed and virtually bioactively screened by docking with a target, then provided by a Friedel–Crafts reaction, esterification (or amidation), and oximation from *p*-substituted phenyl derivatives (Methylbenzene, Methoxybenzene, Chlorobenzene). Anti-hepatitis B virus (HBV) activities of all synthesized compounds were evaluated with HepG2.2.15 cells in vitro. Results showed that most of compounds exhibited low cytotoxicity on HepG2.2.15 cells and significant inhibition on the secretion of HBsAg and HBeAg. Among them, compound **5c-1** showed the most potent activity on inhibiting HBsAg secretion (IC_50_ = 39.93 μM, SI = 28.51). Results of the bioactive screening showed that stronger the compounds bound to target human leukocyte antigen A protein in docking, the more active they were in anti-HBV activities in vitro.

## 1. Introduction

Hepatitis B virus (HBV) infection is a serious worldwide health problem, which causes acute and chronic hepatitis B, cirrhosis, hepatocellular carcinoma, and other hepatic diseases [1]. According to the World Health Organization, 240 million people in the world suffer chronic infection and develop into HBV carriers. About 0.78 million people die per year from HBV-related diseases [2]. The nucleoside analogs used in treatment of anti-HBV play the role of Trojan horse in the synthesis of HBV DNA and suppress replication of HBV [3,4,5,6,7], but they are not effective in eliminating the virus from patients. Meanwhile, HBV therapies with nucleoside analogs in the long term cause serious side effects and resistance [8,9,10,11,12]. For the improvement of HBV therapies, the structural modification of nucleoside analogs has focused and developed many derivatives [13,14,15,16]. Although more and more effective nucleoside analogs agents for treating HBV have been invented and developed, they only inhibit or stop replication of HBV DNA, and do not eliminate HBV cccDNA from patients. The cccDNA in patients will cause HBV DNA replication. Methods to stop and eliminate HBV DNA and cccDNA from HBV patients are still a great challenge for researchers. In searching for more effective anti-HBV agents, many significant anti-HBV non-nucleoside analogs from synthesized compounds [17,18,19,20,21,22,23] and natural products [24,25,26] have been found. Some were designed according to their interactions with receptor by simulating screen [27]. Researchers showed that HLA-A2 with the immunodominant HBcAg18–27 epitope (or HLA-A2.1-restricted CTL epitope) binds peptide of vaccine or of HBcAg to initiate a specific respond of T cell and resolve acute HBV infection [28,29,30]. Some other peptides specifically bound to HBcAg18–27 epitope activate CTL response to prevent infection and eliminate HBV [31,32]. Therefore, this implies that other non-peptide compounds specifically interact with protein residue in HLA-A2.1-restricted CTL epitope may initiate CTL respond to prevent infection by HBV. A protein residue from HLA-A2.1-restricted CTL epitope (PDB ID 3OX8) with intensive interaction with non-nucleoside compounds displayed anti-HBV activities in docking investigations, and this protein residue (3OX8) has been used as a virtual target to design anti-HBV non-nucleoside compounds in our previous work. A series of oxime-containing compounds with a C_6_-C_3_ skeleton were designed, synthesized, and assayed for anti-HBV activities. Results showed that these designed compounds possessed significant anti-HBV activities [33,34,35]. On the basis of these results, we designed new oximes with a C_6_-C_4_ fragment and screened their anti-HBV activities to investigate the structure of the bioactivity relationship. Results of docking studies of these new oxime derivatives showed that N in the oxime group (-O-N=C) and O in the carbonyl group (-C=O) of these compounds interacted with amino acid residue by a hydrogen bond in active site of the HLA-Aprotein (HLA-A*02:03, PDB ID: 3OX8) [36]. This indicated the roles of oxime and carbonyl group in anti-HBV activity in theory.

## 2. Results and Discussion

### 2.1. Molecular Docking Study

**Figure 1:**
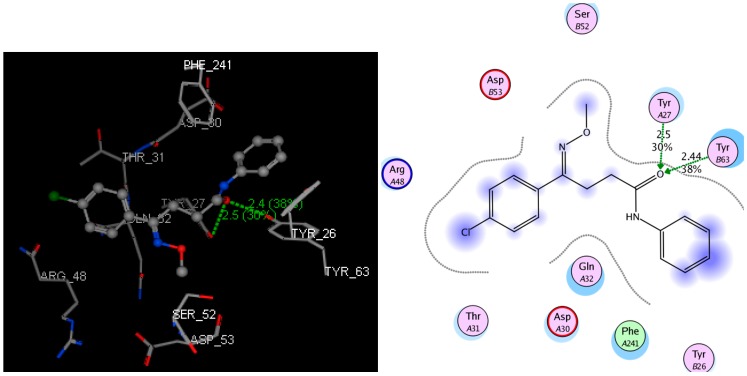
Binding mode of compound **5c-1** with 3OX8. The hydrogen bond formed is colored in green.

**Figure 2:**
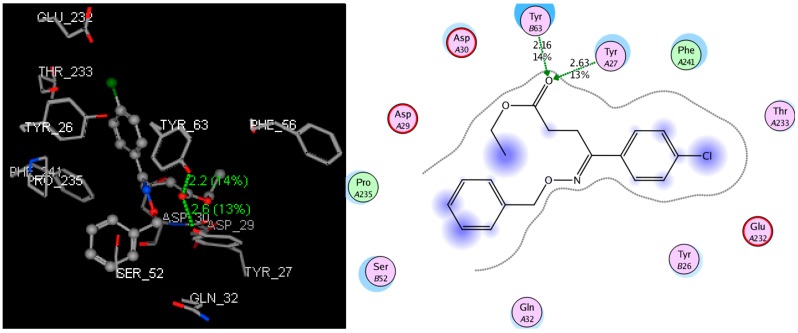
Binding mode of compound **3c-2** with 3OX8. The hydrogen bond formed is colored in green.

**Figure 3:**
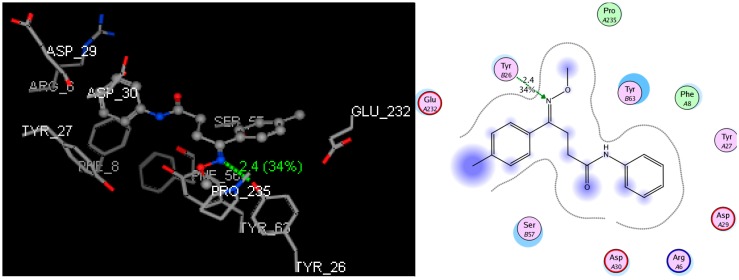
Binding mode of compound **5a-1** with 3OX8. The hydrogen bond formed is colored in green.

Molecular docking studies of the oxime ester derivatives were carried out using MOE 2008.10 as docking software in order to rationalize the biological activity results and understand the various interactions between ligand and protein in the active site in detail. HLA-A protein (HLA-A*02:03, PDB ID: 3OX8) [36] was used for docking study. The “Site Finder” tool of this program was used to search for its active site. Three docking procedures for each ligand were performed and the best configuration of each of the ligand–receptor complexes was selected based on energetic grounds. The affinity-scoring function *δ*G was used to assess and rank the ligand–receptor complexes. The docking scores and the hydrogen bonding strength of all the molecules are shown in Table 1. These oxime ester derivatives had a dock score ranging from −12.0185 to −9.1113, and all of them were involved in at least one hydrogen-bonding interaction with the active site of 3OX8 protein. Among them, N in the oxime group (-O-N=C) of 14 compounds interacted with amino acid residues by a hydrogen bond in the active site, which is similar to the reported works [33]. O in the carbonyl group (-C=O) of five compounds interacted with amino acid residues by a hydrogen bond in the active sites. This indicated in theory the roles of the oxime group and carbonyl group in anti-HBV activity. Bioactive results in vitro showed the compound **5c-1** had the most potent anti-HBV activity with IC_50_ values of 39.93 μM for HBsAg, the next was **5a-1** (HBsAg IC_50_ =74.92 μM), followed by **3c-2** (HBsAg IC_50_ = 94.71 μM, HBeAg IC_50_ = 93.91 μM). Compounds **5c-1** (Figure 1) and **3c-2** (Figure 2) formed two hydrogen bonds in length 2.50 and 2.44 Å, 2.63 and 2.61 Å with O of O=C in amide group of Tyr27 and Tyr63, respectively (Figure 1 and Figure 2), and compound **5a-1** formed only one hydrogen bond in length 2.40 Å with N of C=N in oxime group of Tyr26 (Figure 3).

### 2.2. Chemistry

**Scheme 1:**
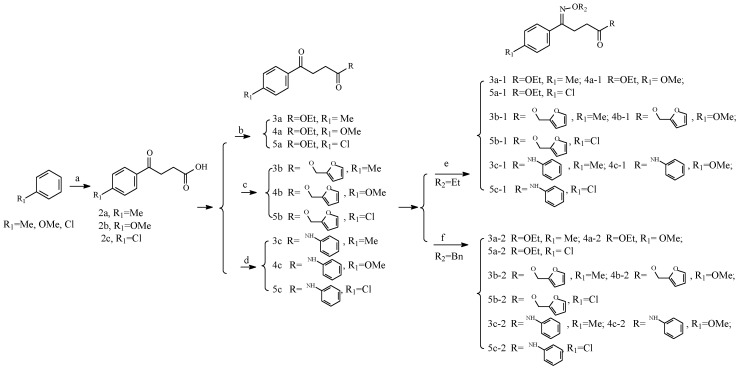
Synthetic route of the series of compounds. Reagents and conditions: (a) succinic anhydride, AlCl_3_/DCM, overnight, r.t.; (b) TsOH/EtOH, refiux, 6–8 h; (c) Furfuryl alcohol, DMAP, DCC/THF, 0.5 h 0 °C; 6–8 h, r.t.; (d) Aniline, DMAP, DCC/THF, 0.5 h 0 °C; 6–8 h, r.t.; (e) NH_2_OCH_3_·HCl, Pyridine/DCM, 70 °C, 6–7 h; (f) NH_2_OCH_2_C_6_H_5_·HCl, Pyridine/DCM, 70 °C, 6–7.

General synthesis of the intermediate and target compounds is depicted in Scheme 1. In the initial step, 3-aroylpropionic acids (**2a**, **2b** and **2c**) were prepared by condensing substituted benzenes with succinic anhydride under Friedel–Crafts acylation reaction conditions [37]. Treatment of 3-aroylpropionic acids (**2a**, **2b** and **2c**) with ethanol in the presence of *p*-toluenesulfonic acid provided ethyl 4-(4-substituted benzoyl)-4-oxobutanoate (**3a**, **3b** and **3c**) in 94~96% yield. Compounds **4a**, **4b**, **4c**, **5a**, **5b** and **5c** were formed by the reaction of 3-aroylpropionic acids (**2a**, **2b** and **2c**) with furfuryl alcohol or aniline in the presence of *N*,*N*-dicyclohexylcarbodiimide and 4-dimethylamioprdine in THF with yields of 88~96% [38,39,40,41,42,43]. The intermediate compounds (**3a**, **3b**, **3c**, **4a**, **4b**, **4c**, **5a**, **5b** and **5c**) finally reacted with methoxylamine or hydroxylamine hydrochloride in pyridine and DCM to afford oxime ethers derivatives (**3a-1**, **3a-2**, **3a-3**; **3b-1**, **3b-2**, **3b-3**; **3c-1**, **3c-2**, **3c-3**; **4a-1**, **4a-2**, **4a-3**; **4b-1**, **4b-2**, **4b-3**; **4c-1**, **4b-2**, **4b-3**; **5a-1**, **5a-2**, **5a-3**; **5b-1**, **5b-2**, **5b-3**; and **5c-1**, **5c-2**, **5c-3**) as mixture of isomers. These isomers were easily separated by column chromatography. The major isomer of oxime ether compounds is presumed to have the *E* configuration [44]. The structures of the synthesized compounds were characterized by ^1^H NMR, ^13^C NMR and MS data and their data are presented in the experimental section. Signals at 2.28~2.35, 3.77~3.84, 3.91~3.98 and 5.17~5.26 ppm in ^1^H NMR were related to CH_3_, OCH_3_, N-OCH_3_, N-OCH_2_ protons, respectively. The chemical shifts of aromatic hydrogens of the phenyl ring appeared in the region *δ* 7.71~6.83. ^13^C NMR spectra of the derivatives showed signals at about 155.61~157.93 and 170.16~172.80 ppm related to carbons in C=N and C=O respectively. The signals at 60.91~62.36 ppm attributed to carbons in N-OCH_3_, and 75.74~76.51 ppm to carbons in N-OCH_2_.

### 2.3. Anti-Hepatitis B Virus (HBV) Activity

**Figure 4:**
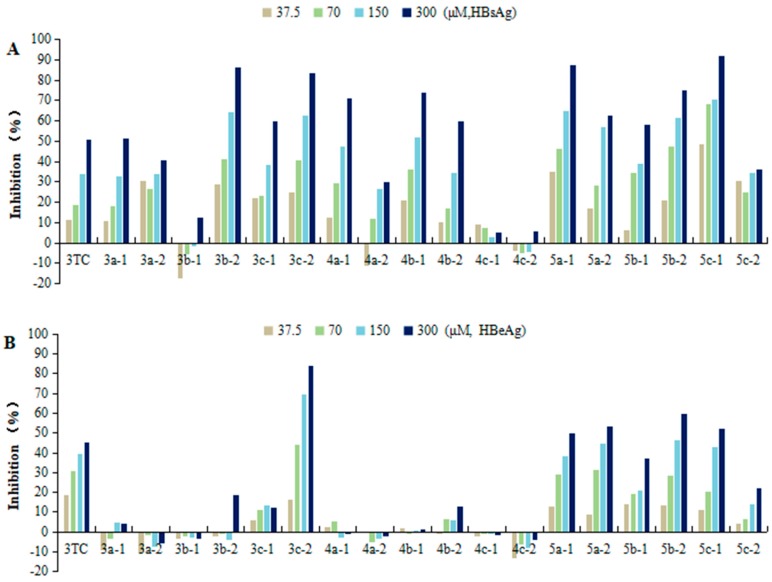
Inhibitory effects of the compounds on secretion of HBsAg (**A**) and HBeAg (**B**) in the HepG 2.2.15 cell line. Date were expressed as mean ± S.D. (*n* = 3).

**Figure 5:**
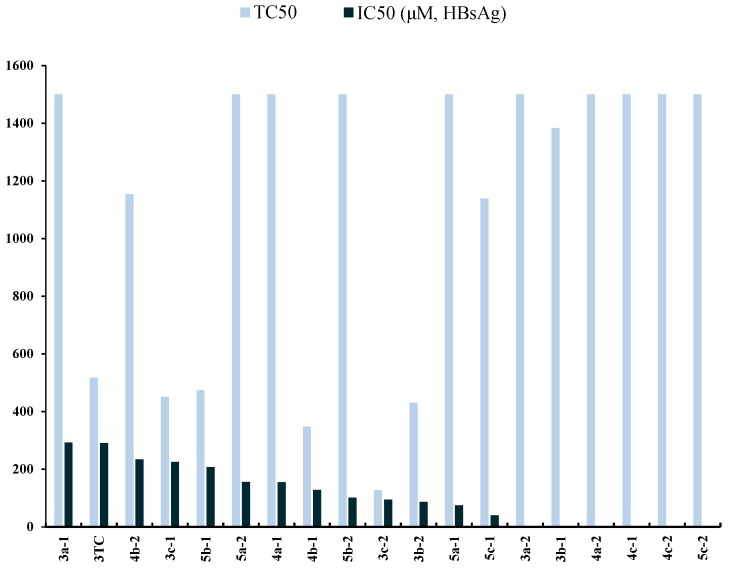
Cytotoxicity and inhibiting HBsAg activity of compounds in 9 days.

**Figure 6:**
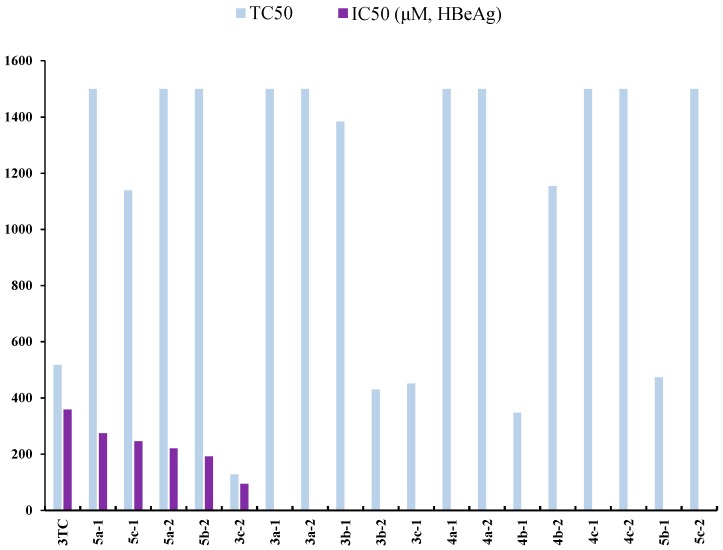
Cytotoxicity and inhibiting HBeAg activity of compounds in 9 days.

All synthesized derivatives were assayed for their anti-HBV activities in vitro, which included inhibiting the secretion of HBsAg and HBeAg in HepG2.2.15 cells with lamivudine (**3TC**, a clinically popular anti-HBV agent) as a positive control. The anti-HBV activities of the compounds were expressed as the concentration of compound that achieved 50% inhibition (IC_50_) to the secretion of HBsAg and HBeAg. Some compounds in which the inhibition rate of the secretion of HBsAg or HBeAg did not reach 50% after **9d** would not have the IC_50_ value calculated (Figure 4A,B). The cytotoxicity of compounds was expressed as the concentration of compound required to kill 50% (TC_50_) of the HepG2.2.15 cells. Parts of compounds were shown to be more active for inhibiting the secretion of HBsAg and had lower cytotoxicity than lamivudine. Eleven among the 18 derivatives displayed higher inhibitory activity against the secretion of HBsAg than lamivudine (Figure 5), and five among those derivatives demonstrated better inhibitory effect in the secretion of HBeAg than lamivudine (Figure 6). Among them, compounds **5c-1** and **5a-1** had the most potential as anti-HBV agents.

### 2.4. Structure–Activity Relationship (SAR)

Most of these compounds showed low cytotoxicity to HepG2.2.15 cells lines except the derivatives **3c-2** and **4b-1**. The inhibition ratio of part of the derivatives on HBeAg was less than 50% in the test concentration range. The anti-HBV activity of the derivatives was evaluated from their inhibition on the secretion of HBsAg. Compounds **5a-1**, **5a-2**, **5b-1**, **5b-2**, **5c-1** and **5c-1** with similar structures but different oxime ether groups showed different anti-HBV activity (Table 2). Compound **5a-1**, with IC_50_ values of 74.92 μM and 273.87 μM for HBsAg and HBeAg, respectively, was shown to be more effective at inhibiting HBsAg secretion but weaker at HBeAg secretion than that of compound **5a-2** (HBsAg IC_50_ = 156.27 μM, HBeAg IC_50_ = 220.09 μM). It was similar to the compounds **5b-1**, **5b-2**, **5c-1** and **5c-1**. Compound **5c-1** (HBeAg IC_50_ = 245.96 μM) was shown to be more effective at inhibiting HBeAg secretion than that of **5c-1** (HBeAg IC_50_ = non). A series of derivative 4 also had similar behavior at inhibiting HBsAg secretion. With an electronic withdrawing group Cl, compound **5a-1** showed a better inhibitory effect on the secretion of HBsAg (IC_50_ = 74.92 μM) than compound **3a-1** and **4a-1** (IC_50_ = 292.73, 154.50 μM). Similar behaviors were shown in compounds **3a-2**, **4a-2** and **5a-2** (IC_50_ = non, non, 156.27 μM) and **3c-1**, **4c-1** and **5c-1**(IC_50_ =224.82, non, 39.93 μM), respectively. This revealed the formation of an amide fragment increased bioactivity. Compared to compound **3b-1,** whose inhibitory activity could be neglected, compound **5b-1** had relatively high inhibitory potency to the secretion of HBsAg (IC_50_ = 207.63 μM). An electronic donating group MeO in compound **4b-1** (with an IC_50_ value of 127.68 μM for HBsAg) was shown to be more effective at inhibiting HBsAg secretion than compound **5b-1**, but appeared toxic (TC_50_ = 347.55 μM, SI = 2.72), but the Me group in **3b-1** presented less bioactivity. As amides, in compounds **3c-1**, **4c-1** and **5c-1** an electronic withdrawing group Cl in **5c-1** presented a more significant inhibiting effect than compounds **3c-1** and **4c-1**. For compounds **3b-2**, **4b-2** and **5b-2**, a more electronic donating group MeO in **4b-2** showed less bioactivity, and compound **5b-2** showed better inhibition of HBsAg secretion (IC_50_ = 101.19 μM) than compound **4b-2** (IC_50_ = 233.60 μM), but weaker than **3b-2** (IC_50_ = 86.85 μM). For amide compounds **3c-2**, **4c-2** and **5c-1**, only a mild electronic donating group Me in **3c-2** showed more bioactivity. In general, most of the compounds with R_2_ = OMe were more bioactive than ther compounds with R_2_ = Bn.

Most *N*-phenyl amide derivatives among all the compounds exhibited an inhibitory effect on HBeAg secretion. These results suggested that the introduction of *N*-phenyl amide shows relatively greater anti-HBV activity than the introduction of furan-2-ylmethyl or ethyl.

According to the results mentioned above, the SARs were summarized. The *N*-phenyl amide-substituted methoxy oxime ether derivatives possessed higher anti-HBV activity than other analogs.

## 3. Methods

### 3.1. Molecular Docking

The docking study was performed using the MOE 2008.10 to understand the ligand–receptor interactions in detail. The crystal structure of human leukocyte antigen (HLA-A) protein (PDB ID: 3OX8) [36] was retrieved from the Protein Data Bank (http://www.rcsb.org/pdb/home/home.do). Conformation of all the compounds was constructed in ChemDraw Ultra 7.0 and optimized in HyperChem 8.0.7 Software, and included hydrogen addition, 3D structure conversion, force-field optimization, and geometry optimization. The optimized ligands were built using the builder interface of the MOE program. The crystal structure was imported into MOE and chain A was considered for the docking process as the protein is a dimer consisting of A and B chains. The structure was protonated, unbound waters were deleted, polar hydrogens were added, and energy minimization was carried out. The active site was correlated with the ‘Site Finder’ module of MOE to define the docking site for the ligands [33,34,35]. The docking procedure was followed using the standard protocol implemented in MOE 2008.10 and the geometry of the resulting complexes was studied using the MOE’s Pose Viewer utility.

### 3.2. Synthesis Methods

#### 3.2.1. Materials and Methods

Melting points were determined using a fully automatic melting point apparatus MP420 (Jinan, China) and were uncorrected. Mass spectrometry (MS) spectra ware recorded on Thermo Scientific ITQ 1100 instrument (Thermo Fisher Scientific, Waltham, MA, USA), Varian CP 3800^+^ Satum 2200 instrument (Agilent Technologies, Santa Clara, CA, USA). NMR spectra were recorded on a Bruker AV III HD 600 MHz (^1^H/^13^C, 600 MHz/150 MHz) spectrometer (Brucker, Corp, MA, USA) by using CDCl_3_, CD_3_OH, or DMSO as solvent and TMS as an internal standard. All the chemicals were obtained from local suppliers and were used without further purification.

#### 3.2.2. Chemistry 

The general procedure (Scheme 1) for the preparation of compounds (**2a**, **2b** and **2c**): succinic anhydride (1 equiv, 10 mmol) was reacted with an appropriate aromatic compound (substituted benzene, 1 equiv, 10 mmol) in DCM (20 mL) in the presence of anhydrous aluminium chloride (1.5 equiv, 15 mmol). The reaction mixture was stirred under anhydrous conditions overnight at room temperature and then ice-cold diluted hydrochloric acid solution was added dropwise. A solid mass separated out which was filtered and purified by recrystallization to give **2a**, **2b** and **2c** [44].

The general procedure for the preparation of compounds (**3a**, **3b** and **3c**): a mixture of the appropriate acid **2a-c** (1 equiv, 10 mmol) and *p*-toluenesulfonic acid (0.4 equiv, 4 mmol) in EtOH (20 mL) was refluxed for 6–8 h and evaporated to remove EtOH. The residue was suspended in H_2_O (30 mL) and extracted with EtOAc (2 × 50 mL) which was further purified by column chromatography on silica gel eluting with AcOEt–petroleum ether (1:4, *v*/*v*) to get **3a-c** as crystals. The general procedure for the preparation of compounds (**4a-c**–**5a-c**): an appropriate acid **2a-b** (1 equiv, 10 mmol), and 4-dimethylamioprdine (0.02 equiv, 0.2 mmol) was added to a solution of the furfuryl alcohol or aniline (1 equiv, 10 mmol) in THF (20 mL). The mixture was stirred and cooled to 0 °C and then *N*,*N*-dicyclohexylcarbodiimide (DCC) (1.1 equiv, 11 mmol) was added over a 5-min period and the reaction was stirred under anhydrous conditions for 6–8 h at room temperature. The mixture was filtered and the filtrate was evaporated to yield a crude product which was finally purified by recrystallization to give **4a**, **4b**, **4c**, **5a**, **5b** and **5c** as crystals [38,39,40,41,42,43].

*3-(4-Methylbenzoyl)propionic acid* (**2a**). Yield 95%; m.p. 93.7~94.9 °C. ^1^H NMR (600 MHz, MeOD) *δ*: 7.91–7.86 (m, 2H, H-6, 10), 7.29 (d, *J* = 8.0 Hz, 2H, H-7, 9), 3.30–3.25 (m, 2H, H-3), 2.70–2.66 (m, 2H, H-2), 2.39 (s, 3H, H-11).

*3-(4-Methoxybenzoyl)propionic acid* (**2b**). Yield 98%; m.p. 148.9~150.2 °C. ^1^H NMR (600 MHz, MeOD) *δ*: 7.90–7.85 (m, 2H, H-6, 10), 6.92–6.87 (m, 2H, H-7, 9), 3.76 (s, 3H, H-11), 3.18–3.14 (m, 2H, H-3), 2.60–2.56 (m, 2H, H-2).

*3-(4-Chlorobenzoyl)propionic acid* (**2c**). Yield 90%; m.p. 130.2~131.5 °C. ^1^H NMR (600 MHz, DMSO) *δ*: 12.16 (s, 1H, H-OH), 7.99 (dd, *J* = 8.5, 1.7 Hz, 2H, H-6, 10), 7.62–7.58 (m, 2H, H-7, 9), 3.26–3.22 (m, 2H, H-3), 2.58 (t, *J* = 6.2 Hz, 2H H-2).

*Ethyl 4-(4-methylbenzoyl)-4-oxobutanoate* (**3a**). Yield 96%; m.p. 66.6~68.0 °C. ^1^H NMR (600 MHz, MeOD) *δ*: 7.88 (dd, *J* = 8.2, 1.7 Hz, 2H, H-6, 10), 7.29 (d, *J* = 6.8 Hz, 2H, H-7, 9), 4.11 (q, *J* = 7.1 Hz, 2H, H-1′), 3.29 (td, *J* = 6.5, 2.3 Hz, 2H, H-3), 2.70–2.66 (m, 2H, H-2), 2.39 (s, 3H, H-11), 1.23 (t, *J* = 7.1 Hz, 3H, H-2′).

*Ethyl 4-(4-methoxyphenyl)-4-oxobutanoate* (**3b**). Yield 98%; m.p. 55.9~57.1 °C. ^1^H NMR (600 MHz, MeOD) *δ*: 7.90–7.85 (m, 2H, H-6, 10), 6.92–6.87 (m, 2H, H-7, 9), 4.02 (q, *J* = 7.1 Hz, 2H, H-1′), 3.76 (s, 3H, H-11), 3.19–3.16 (m, 2H, H-3), 2.58 (dd, *J* = 7.3, 5.4 Hz, 2H, H-2), 1.13 (t, *J* = 7.1 Hz, 3H, H-2′).

*Ethyl 4-(4-chlorophenyl)-4-oxobutanoate* (**3c**). Yield 94%; m.p. 55.5~56.5 °C. ^1^H NMR (600 MHz, CDCl_3_) *δ*: 7.93 (t, *J* = 5.4 Hz, 2H, H-6, 10), 7.44 (t, *J* = 5.4 Hz, 2H, H-7, 9), 4.16 (q, *J* = 7.1 Hz, 2H, H-1′), 3.27 (t, *J* = 6.6 Hz, 2H, H-3), 2.75 (t, *J* = 6.6 Hz, 2H, H-2), 1.27 (t, *J* = 7.1 Hz, 3H, H-2′).

*Furan-2-ylmethyl-4-(4-methylbenzoyl)-4- oxobutanoate* (**4a**). Yield 95%, m.p. 79.6~80.5 °C; ^1^H NMR (600 MHz, CDCl_3_) *δ*: 7.87 (d, *J* = 8.2 Hz, 2H, H-6, 10), 7.41 (d, *J* = 1.1 Hz, 1H, H-5′), 7.25 (d, *J* = 8.1 Hz, 2H, H-7, 9), 6.40 (d, *J* = 3.2 Hz, 1H, H-3′), 6.36 (dd, *J* = 3.1, 1.9 Hz, 1H, H-4′), 5.09 (s, 2H, H-1′), 3.29 (t, *J* = 6.7 Hz, 2H, H-3), 2.78 (t, *J* = 6.7 Hz, 2H, H-2), 2.41 (s, 3H, H-11); ^13^C NMR (151 MHz, CDCl_3_) *δ*: 197.56 (C-4), 172.66 (C-1), 149.45 (C-2′), 144.03 (C-8), 143.24 (C-5′), 134.07 (C-5), 129.29 (C-7, 9), 128.16 (C-6, 10), 110.63 (C-4′), 110.56 (C-3′), 58.28 (C-1′), 33.19 (C-3), 28.19 (C-2), 21.65 (C-11); ESIMS: *m*/*z* 273 [M + H]^+^, 290 [M + NH_4_]^+^, 295 [M + Na]^+^, 567 [2M + Na]^+^, calc. for C_16_H_16_O_4_ (272.2958).

*Furan-2-ylmethyl 4-(4-methoxyphenyl)-4-oxobutanoate* (**4b**). Yield 96%, m.p. 61.0~61.7 °C; ^1^H NMR (600 MHz, CDCl_3_) *δ*: 7.97–7.94 (m, 2H, H-6, 10), 7.43–7.40 (m, 1H, H-5′), 6.96–6.91 (m, 2H, H-7, 9), 6.40 (d, *J* = 3.2 Hz, 1H, H-3′), 6.36 (dd, *J* = 3.1, 1.9 Hz, 1H, H-4′), 5.09 (s, 2H, H-1′), 3.86 (s, 3H, H-11), 3.27 (t, *J* = 6.7 Hz, 2H, H-3), 2.78 (t, *J* = 6.7 Hz, 2H, H-2); ^13^C NMR (151 MHz, CDCl_3_) *δ*: 196.44 (C-4), 172.72 (C-1), 163.59 (C-8), 149.46 (C-2′), 143.24 (C-5′), 130.31 (C-6, 10), 129.65 (C-5), 113.75 (C-7, 9), 110.62 (C-4′), 110.57 (C-3′), 58.27 (C-1′), 55.47 (C-11), 32.93 (C-3), 28.25 (C-2); ESIMS: *m*/*z* 289 [M + H]^+^, 311 [M + Na]^+^, calc. for C_16_H_16_O_5_ (288.2952).

*Furan-2-ylmethyl 4-(4-chlorophenyl)-4-oxobutanoate* (**4c**). Yield 90%, m.p. 64.2~65.1 °C; ^1^H NMR (600 MHz, CDCl_3_) *δ*: 7.91 (d, *J* = 8.5 Hz, 2H, H-6, 10), 7.44 (d, *J* = 8.5 Hz, 2H, H-7, 9), 7.42 (s, 1H, H-5′), 6.41 (d, *J* = 3.2 Hz, 1H, H-3′), 6.36 (d, *J* = 1.8 Hz, 1H, H-4′), 5.09 (s, 2H, H-1′), 3.28 (t, *J* = 6.6 Hz, 2H, H-3), 2.80 (t, *J* = 6.6 Hz, 2H, H-2); ^13^C NMR (151 MHz, CDCl_3_) *δ*: 196.76 (C-4), 172.43 (C-1), 149.34 (C-2′), 143.29 (C-5′), 139.70 (C-8), 134.86 (C-5), 129.47 (C-6, 10), 128.95 (C-7, 9), 110.70 (C-4′), 110.58 (C-3′), 58.35 (C-1′), 33.26 (C-3), 28.08 (C-2); ESIMS: *m*/*z* 293 [M + H]^+^, 310 [M + NH_4_]^+^, 315 [M + Na]^+^, calc. for C_15_H_13_ClO_4_ (292.7143).

*4-(4-Methylbenzoyl)-4-oxo-N-phenylbutanamide* (**5a**). Yield 90%, m.p. 147.5~148.5 °C; ^1^H NMR (600 MHz, DMSO) *δ*: 10.04 (s, 1H, H-NH), 7.91 (d, *J* = 8.2 Hz, 2H, H-6, 10), 7.60 (t, *J* = 8.4 Hz, 2H, H-2′, 6′), 7.34 (d, *J* = 7.1 Hz, 2H, H-7, 9), 7.29 (t, *J* = 7.9 Hz, 2H, H-3′, 5′), 7.02 (t, *J* = 7.4 Hz, 1H, H-4′), 3.31 (t, *J* = 6.4 Hz, 2H, H-3), 2.72 (t, *J* = 6.3 Hz, 2H, H-2), 2.38 (s, 3H, H-11); ^13^C NMR (151 MHz, DMSO) *δ*: 198.80 (C-4), 170.84 (C-1), 143.94 (C-8), 139.83 (C-1′), 134.55 (C-5), 129.70 (C-7, 9), 129.11 (C-3′, 5′), 128.43 (C-6, 10), 123.33 (C-4′), 119.35 (C-2′, 6′), 33.46 (C-3), 30.76 (C-2), 21.61 (C-11); ESIMS: *m*/*z* 268 [M + H]^+^, calc. for C_17_H_17_NO_2_ (267.3224).

*4-(4-Methoxyphenyl)-4-oxo-N-phenylbutanamide* (**5b**). Yield 93%, m.p. 113.3~114.2 °C; ^1^H NMR (600 MHz, DMSO) *δ*: 10.01 (s, 1H, H-NH), 8.00–7.96 (m, 2H, H-6, 10), 7.59 (d, *J* = 7.6 Hz, 2H, H-2′, 6′), 7.30–7.26 (m, 2H, H-3′, 5′), 7.07–7.03 (m, 2H, H-7, 9), 7.01 (t, *J* = 7.4 Hz, 1H, H-4′), 3.85 (s, 3H, H-11), 3.28 (t, *J* = 6.5 Hz, 2H, H-3), 2.70 (t, *J* = 6.5 Hz, 2H, H-2); ^13^C NMR (151 MHz, DMSO) *δ*: 197.64 (C-4), 170.90 (C-1), 163.56 (C-8), 139.87 (C-1′), 130.64 (C-6, 10), 130.00 (C-5), 129.12 (C-3′, 5′), 123.31 (C-4′), 119.34 (C-2′, 6′), 114.34 (C-7, 9), 55.99 (C-11), 33.22 (C-3), 30.83 (C-2); ESIMS: *m*/*z* 284 [M + H]^+^, calc. for C_17_H_17_NO_3_ (283.3218).

*4-(4-Chlorophenyl)-4-oxo-N-phenylbutanamide* (**5c**). Yield 88%, m.p. 140.2~141.0 °C; ^1^H NMR (600 MHz, DMSO) *δ*: 10.04 (s, 1H, H-NH), 8.03–8.00 (m, 2H, H-6, 10), 7.60 (dd, *J* = 12.5, 5.5 Hz, 4H, H-2′, 6′, 7, 9), 7.28 (t, *J* = 7.9 Hz, 2H, H-3′, 5′), 7.02 (t, *J* = 7.4 Hz, 1H, H-4′), 3.33 (t, *J* = 6.4 Hz, 2H, H-3), 2.73 (t, *J* = 6.4 Hz, 2H, H-2); ^13^C NMR (151 MHz, MeOD) *δ*: 198.08 (C-4), 171.77 (C-1), 139.13 (C-8), 138.58 (C-1′), 135.26 (C-5), 129.41 (C-6, 10), 128.56 (C-3′, 5′), 128.37 (C-7, 9), 123.62 (C-4′), 119.76 (C-2′, 6′), 33.17 (C-3), 30.05 (C-2); ESIMS: *m*/*z* 288 [M + H]^+^, calc. for C_16_H_14_ClNO_2_ (287.7409).

*Ethyl (E)-4-(4-methylbenzoyl)-4-[(methoxy)imino]butanoate* (**3a-1**). Yield 78%; ^1^H NMR (600 MHz, MeOD) *δ*: 7.52 (dd, *J* = 4.9, 3.3 Hz, 2H, H-6, 10), 7.22–7.17 (m, 2H, H-7, 9), 4.07 (dd, *J* = 12.7, 5.5 Hz, 2H, H-1′), 3.95 (s, 3H, H-1″), 3.04–2.98 (m, 2H, H-3), 2.50 (ddd, *J* = 7.8, 4.1, 1.5 Hz, 2H, H-2), 2.34 (s, 3H, H-11), 1.20 (t, *J* = 7.1 Hz, 3H, H-2′); ^13^C NMR (151 MHz, MeOD) *δ*: 172.80 (C-1), 156.95 (C-4), 139.20 (C-8), 132.18 (C-5), 128.81 (C-7, 9), 125.99 (C-6, 10), 60.91 (C-1″), 60.34 (C-1′), 30.59 (C-2), 21.83 (C-3), 19.93 (C-11), 13.09 (C-2′); ESIMS: *m*/*z* 250.1442 [M + H]^+^, 272.1261 [M + Na]^+^, calc. for C_14_H_19_NO_3_ (249.3056).

*Ethyl (E)-4-(4-methylbenzoyl)-4-[(benzyloxy)imino]butanoate* (**3a-2**). Yield 76%; ^1^H NMR (600 MHz, CDCl_3_) *δ*: 7.52 (d, *J* = 8.1 Hz, 2H, H-6, 10), 7.40 (d, *J* = 7.3 Hz, 2H, H-3″, 7″), 7.35 (t, *J* = 7.5 Hz, 2H, H-4″, 6″), 7.30 (t, *J* = 7.2 Hz, 1H, H-5″), 7.16 (d, *J* = 8.0 Hz, 2H, H-7, 9), 5.22 (s, 2H, H-1″), 4.08 (q, *J* = 7.1 Hz, 2H, H-1′), 3.06 (dd, *J* = 10.5, 5.8 Hz, 2H, H-3), 2.57–2.52 (m, 2H, H-2), 2.35 (s, 3H, H-11), 1.20 (t, *J* = 7.1 Hz, 3H, H-2′); ^13^C NMR (151 MHz, MeOD) *δ*: 172.77 (C-1), 157.28 (C-4), 139.20 (C-8), 138.02 (C-2″), 132.20 (C-5), 128.80 (C-7, 9), 128.03 (C-3″, 7″), 127.84 (C-4″, 6″), 127.48 (C-5″), 126.04 (C-6, 10), 75.86 (C-1″), 60.34 (C-1′), 30.58 (C-2), 22.02 (C-3), 20.01 (C-11), 13.08 (C-2′); ESIMS: *m*/*z* 326.1766 [M + H]^+^, 348.1579 [M + Na]^+^, calc. for C_20_H_23_NO_3_ (325.4015).

*Ethyl (E)-4-(4-methoxyphenyl)-4-[(methoxy)imino]butanoate* (**3b-1**). Yield 76%; ^1^H NMR (600 MHz, CDCl_3_) *δ*: 7.60–7.57 (m, 2H, H-6, 10), 6.91–6.87 (m, 2H, H-7, 9), 4.11 (q, *J* = 7.1 Hz, 2H, H-1′), 3.96 (s, 3H, H-1″), 3.82 (s, 3H, H-11), 3.03–2.99 (m, 2H, H-3), 2.56–2.52 (m, 2H, H-2), 1.23 (t, *J* = 7.1 Hz, 3H, H-2′); ^13^C NMR (151 MHz, CDCl_3_) *δ*: 172.73 (C-1), 160.52 (C-8), 156.46 (C-4), 127.67 (C-5), 127.64 (C-6, 10), 113.94 (C-7, 9), 61.91 (C-1″), 60.59 (C-1′), 55.32 (C-11), 30.99 (C-2), 22.28 (C-3), 14.18 (C-2′); ESIMS: *m*/*z* 266.1389 [M + H]^+^, 288.1199 [M + Na]^+^, calc. for C_14_H_19_NO_4_ (265.3050).

*Ethyl (E)-4-(4-methoxyphenyl)-4-[(benzyloxy)imino]butanoate* (**3b-2**). Yield 78%; ^1^H NMR (600 MHz, CDCl_3_) *δ*: 7.66–7.62 (m, 2H, H-6, 10), 7.45 (d, *J* = 7.3 Hz, 2H, H-3″, 7″), 7.40 (t, *J* = 7.5 Hz, 2H, H-4″, 6″), 7.34 (t, *J* = 7.3 Hz, 1H, H-5″), 6.94–6.90 (m, 2H, H-7, 9), 5.26 (s, 2H, H-1″), 4.13 (q, *J* = 7.1 Hz, 2H, H-1′), 3.84 (s, 3H, H-11), 3.14–3.07 (m, 2H, H-3), 2.63–2.57 (m, 2H, H-2), 1.25 (t, *J* = 7.2 Hz, 3H, H-2′); ^13^C NMR (151 MHz, CDCl_3_) *δ*: 172.72 (C-1), 160.57 (C-8), 156.76 (C-4), 138.11 (C-2″), 128.38 (C-3″, 7″), 128.11 (C-4″, 6″), 127.76 (C-5″), 127.72 (C-6), 127.68 (C-5), 113.93 (C-7, 9), 76.22 (C-1″), 60.60 (C-1′), 55.32 (C-11), 30.99 (C-2), 22.40 (C-3), 14.19 (C-2′); ESIMS: *m*/*z* 342.1694 [M + H]^+^, 364.1506 [M + Na]^+^, calc. for C_20_H_23_NO_4_ (341.4009).

*Ethyl (E)-4-(4-chlorophenyl)-4-[(methoxy)imino]butanoate* (**3c-1**). Yield 75%; ^1^H NMR (600 MHz, CDCl_3_) *δ*: 7.60–7.56 (m, 2H, H-6, 10), 7.35–7.31 (m, 2H, H-7, 9), 4.10 (q, *J* = 7.2 Hz, 2H, H-1′), 3.98 (s, 3H, H-1″), 3.02–2.98 (m, 2H, H-3), 2.55–2.52 (m, 2H, H-2), 1.22 (t, *J* = 7.2 Hz, 3H, H-2′); ^13^C NMR (151 MHz, CDCl_3_) *δ*: 172.46 (C-1), 155.74 (C-4), 135.21 (C-8), 133.66 (C-5), 128.71 (C-7, 9), 127.55 (C-6, 10), 62.16 (C-1″), 60.65 (C-1′), 30.77 (C-2), 22.12 (C-3), 14.15 (C-2′); ESIMS: *m*/*z* 270.0901 [M + H]^+^, 292.0720 [M + Na]^+^, calc. for C_13_H_16_ClNO_3_ (269.7240).

*Ethyl (E)-4-(4-chlorophenyl)-4-[(benzyloxy)imino]butanoate* (**3c-2**). Yield 77%; ^1^H NMR (600 MHz, CDCl_3_) *δ*: 7.59–7.56 (m, 2H, H-6, 10), 7.41–7.34 (m, 4H, H-3″, 7″, 4″, 6″), 7.34–7.29 (m, 3H, H-7, 9, 5″), 5.22 (s, 2H, H-1″), 4.08 (q, *J* = 7.1 Hz, 2H, H-1′), 3.06–3.02 (m, 2H, H-3), 2.56–2.52 (m, 2H, H-2), 1.20 (t, *J* = 7.1 Hz, 3H, H-2′); ^13^C NMR (151 MHz, CDCl_3_) *δ*: 172.47 (C-1), 156.11 (C-4), 137.74 (C-2″), 135.26 (C-8), 133.65 (C-5), 128.69 (C-7, 9), 128.40 (C-3″, 7″), 128.12 (C-4″, 6″), 127.87 (C-5″), 127.62 (C-6), 76.49 (C-1″), 60.66 (C-1′), 30.75 (C-2), 22.26 (C-3), 14.14 (C-2′); ESIMS: *m*/*z* 346.1204 [M + H]^+^, 368.1020 [M + Na]^+^, calc. for C_19_H_20_ClNO_3_ (345.8200).

*Furan-2-ylmethyl (E)-4-(4-methylbenzoyl)-4-[(methoxy)imino] butanoate* (**4a-1**). Yield 76%; ^1^H NMR (600 MHz, CDCl_3_) *δ*: 7.50 (d, *J* = 8.1 Hz, 2H, H-6, 10), 7.41 (dd, *J* = 1.8, 0.8 Hz, 1H, H-5′), 7.16 (d, *J* = 8.0 Hz, 2H, H-7, 9), 6.39 (d, *J* = 3.1 Hz, 1H, H-3′), 6.35 (dd, *J* = 3.2, 1.9 Hz, 1H, H-4′), 5.04 (s, 2H, H-1′), 3.95 (s, 3H, H-1″), 3.05–3.00 (m, 2H, H-3), 2.60–2.55 (m, 2H, H-2), 2.35 (s, 3H, H-11); ^13^C NMR (151 MHz, CDCl_3_) *δ*: 172.29 (C-1), 156.63 (C-4), 149.39 (C-2′), 143.27 (C-5′), 139.27 (C-8), 132.25 (C-5), 129.25 (C-7, 9), 126.16 (C-6, 10), 110.68 (C-4′), 110.57 (C-3′), 61.96 (C-1″), 58.18 (C-1′), 30.72 (C-2), 22.22 (C-3), 21.25 (C-11); ESIMS: *m*/*z* 302 [M + H]^+^, calc. for C_17_H_19_NO_4_ (301.3371).

*Furan-2-ylmethyl (E)-4-(4-methylbenzoyl)-4-[(benzyloxy)imino]butanoate* (**4a-2**). Yield 77%; ^1^H NMR (600 MHz, CDCl_3_) *δ*: 7.50 (d, *J* = 8.2 Hz, 2H, H-6, 10), 7.41–7.40 (m, 1H, H-5′), 7.38 (d, *J* = 7.3 Hz, 2H, H-3″, 7″), 7.34 (t, *J* = 7.4 Hz, 2H, H-4″, 6″), 7.31–7.27 (m, 1H, H-5″), 7.15 (d, *J* = 8.0 Hz, 2H, H-7, 9), 6.36 (d, *J* = 3.2 Hz, 1H, H-3′), 6.35 (dd, *J* = 3.2, 1.9 Hz, 1H, H-4′), 5.20 (s, 2H, H-1″), 5.01 (s, 2H, H-1′), 3.09–3.04 (m, 2H, H-3), 2.60–2.55 (m, 2H, H-2), 2.34 (s, 3H, H-11); ^13^C NMR (151 MHz, CDCl_3_) *δ*: 172.28 (C-1), 156.94 (C-4), 149.38 (C-2′), 143.25 (C-5′), 139.31 (C-8), 137.97 (C-2″), 132.24 (C-5), 129.22 (C-7, 9), 128.35 (C-3″, 7″), 128.10 (C-4″, 6″), 127.74 (C-5″), 126.22 (C-6, 10), 110.66 (C-4′), 110.55 (C-3′), 76.25 (C-1″), 58.17 (C-1′), 30.69 (C-2), 22.32 (C-3), 21.25 (C-11); ESIMS: *m*/*z* 378 [M + H]^+^, 400 [M + Na]^+^, calc. for C_23_H_23_NO_4_ (377.4330).

*Furan-2-ylmethyl (E)-4-(4-methoxyphenyl)-4-[(methoxy)imino]butanoate* (**4b-1**). Yield 77%; ^1^H NMR (600 MHz, CDCl_3_) *δ*: 7.60–7.54 (m, 2H, H-6, 10), 7.41 (dd, *J* = 1.8, 0.8 Hz, 1H, H-5′), 6.90–6.85 (m, 2H, H-7, 9), 6.39 (d, *J* = 3.1 Hz, 1H, H-3′), 6.36 (dd, *J* = 3.2, 1.9 Hz, 1H, H-4′), 5.04 (s, 2H, H-1′), 3.94 (s, 3H, H-1″), 3.81 (s, 3H, H-11), 3.04–2.98 (m, 2H, H-3), 2.60–2.56 (m, 2H, H-2); ^13^C NMR (151 MHz, CDCl_3_) *δ*: 172.33 (C-1), 160.51 (C-8), 156.29 (C-4), 149.39 (C-2′), 143.28 (C-5′), 127.63 (C-6, 10), 127.58 (C-5), 113.94 (C-7, 9), 110.70 (C-4′), 110.57 (C-3′), 61.91 (C-1″), 58.20 (C-1′), 55.31 (C-11), 30.76 (C-2), 22.19 (C-3); ESIMS: *m*/*z* 318 [M + H]^+^, calc. for C_17_H_19_NO_5_ (317.3365).

*Furan-2-ylmethyl (E)-4-(4-methoxyphenyl)-4-[(benzyloxy)imino]butanoate* (**4b-2**). Yield 78%; ^1^H NMR (600 MHz, CDCl_3_) *δ*: 7.61–7.53 (m, 2H, H-6, 10), 7.40 (s, 1H, H-5′), 7.38 (d, *J* = 7.7 Hz, 2H, H-3″, 7″), 7.34 (dd, *J* = 10.9, 4.0 Hz, 2H, H-4″, 6″), 7.29 (t, *J* = 7.1 Hz, 1H, H-5″), 6.91–6.83 (m, 2H, H-7, 9), 6.37 (d, *J* = 2.8 Hz, 1H, H-3′), 6.36–6.31 (m, 1H, H-4′), 5.19 (s, 2H, H-1″), 5.01 (s, 2H, H-1′), 3.80 (s, 3H, H-11), 3.11–3.00 (m, 2H, H-3), 2.63–2.53 (m, 2H, H-2); ^13^C NMR (151 MHz, CDCl_3_) *δ*: 172.30 (C-1), 160.53 (C-8), 156.56 (C-4), 149.37 (C-2′), 143.26 (C-5′), 138.03 (C-2″), 128.34 (C-3″, 7″), 128.08 (C-4″, 6″), 127.72 (C-5″), 127.69 (C-6, 10), 127.57 (C-5), 113.90 (C-7, 9), 110.67 (C-4′), 110.56 (C-3′), 76.19 (C-1″), 58.18 (C-1′), 55.30 (C-11), 30.73 (C-2), 22.27 (C-3); ESIMS: *m*/*z* 394 [M + H]^+^, calc. for C_23_H_23_NO_5_ (393.4324).

*Furan-2-ylmethyl (E)-4-(4-chlorophenyl)-4-[(methoxy)imino]butanoate* (**4c-1**). Yield 75%; ^1^H NMR (600 MHz, CDCl_3_) *δ*: 7.58–7.54 (m, 2H, H-6, 10), 7.41 (dd, *J* = 1.7, 0.6 Hz, 1H, H-5′), 7.34–7.30 (m, 2H, H-7, 9), 6.38 (d, *J* = 3.2 Hz, 1H, H-3′), 6.36 (dd, *J* = 3.2, 1.9 Hz, 1H, H-4′), 5.04 (s, 2H, H-1′), 3.96 (s, 3H, H-1″), 3.03–2.99 (m, 2H, H-3), 2.60–2.56 (m, 2H, H-2); ^13^C NMR (151 MHz, CDCl_3_) *δ*: 172.11 (C-1), 155.61 (C-4), 149.28 (C-2′), 143.32 (C-5′), 135.24 (C-8), 133.58 (C-5), 128.73 (C-7, 9), 127.55 (C-6, 10), 110.77 (C-4′), 110.58 (C-3′), 62.18 (C-1″), 58.24 (C-1′), 30.59 (C-2), 22.07 (C-3); ESIMS: *m*/*z* 322 [M + H]^+^, calc. for C_16_H_16_ClNO_4_ (321.7555).

*Furan-2-ylmethyl (E)-4-(4-chlorophenyl)-4-[(benzyloxy)imino]butanoate* (**4c-2**). Yield 76%; ^1^H NMR (600 MHz, CDCl_3_) *δ*: 7.55 (dt, *J* = 4.3, 1.8 Hz, 2H, H-6, 10), 7.41 (s, 1H, H-5′), 7.40–7.37 (m, 2H, H-3″, 7″), 7.37–7.33 (m, 2H, H-4″, 6″), 7.33–7.28 (m, 3H, H-7, 9, 5″), 6.36 (s, 1H, H-3′), 6.35 (dd, *J* = 3.2, 1.7 Hz, 1H, H-4′), 5.21 (s, 2H, H-1″), 5.01 (s, 2H, H-1′), 3.04 (dd, *J* = 11.7, 4.2 Hz, 2H, H-3), 2.61–2.56 (m, 2H, H-2); ^13^C NMR (151 MHz, CDCl_3_) *δ*: 172.09 (C-1), 155.95 (C-4), 149.26 (C-2′), 143.30 (C-5′), 137.69 (C-2″), 135.27 (C-8), 133.57 (C-5), 128.70 (C-7, 9), 128.41 (C-3″, 7″), 128.14 (C-4″, 6″), 127.88 (C-5″), 127.61 (C-6, 10), 110.75 (C-4′), 110.57 (C-3′), 76.51 (C-1″), 58.23 (C-1′), 30.57 (C-2), 22.17 (C-3); ESIMS: *m*/*z* 398 [M + H]^+^, calc. for C_22_H_20_ClNO_4_ (397.8515).

*(E)-4-(4-Methylbenzoyl)-4-[(methoxy)imino]-N-phenylbutanamide* (**5a-1**). Yield 78%, m.p. 94.4~95.3 °C; ^1^H NMR (600 MHz, DMSO) *δ*: 9.91 (s, 1H, H-NH), 7.58 (t, *J* = 8.4 Hz, 4H, H-6, 10, 2′, 6′), 7.31–7.26 (m, 2H, H-3′, 5′), 7.22 (d, *J* = 8.0 Hz, 2H, H-7, 9), 7.02 (t, *J* = 7.4 Hz, 1H, H-4′), 3.91 (s, 3H, H-1″), 3.01–2.96 (m, 2H, H-3), 2.52–2.48 (m, 2H, H-2), 2.32 (s, 3H, H-11); ^13^C NMR (151 MHz, DMSO) *δ*: 170.26 (C-1), 157.46 (C-4), 139.62 (C-1′), 139.30 (C-8), 132.45 (C-5), 129.58 (C-7, 9), 129.12 (C-3′, 5′), 126.56 (C-6, 10), 123.53 (C-4′), 119.56 (C-2′, 6′), 62.12 (C-1″), 33.40 (C-2), 22.43 (C-3), 21.27 (C-11); ESIMS: *m*/*z* 297.1607 [M + H]^+^, 319.1411 [M + Na]^+^, calc. for C_18_H_20_N_2_O_2_ (296.3636).

*(E)-4-(4-Methylbenzoyl)-4-[(benzyloxy)imino]-N-phenylbutan amide* (**5a-2**). Yield 79%, m.p. 119.5~120.5 °C; ^1^H NMR (600 MHz, DMSO) *δ*: 9.95 (s, 1H, H-NH), 7.53 (dd, *J* = 7.5, 5.1 Hz, 4H, H-6, 10, 2′, 6′), 7.38 (d, *J* = 7.4 Hz, 2H, H-3″, 7″), 7.34 (t, *J* = 7.4 Hz, 2H, H-4″, 6″), 7.30–7.25 (m, 3H, H-3′, 5′, 5″), 7.19 (d, *J* = 7.9 Hz, 2H, H-7, 9), 7.03 (t, *J* = 7.4 Hz, 1H, H-4′), 5.17 (s, 2H, H-1″), 3.06–2.98 (m, 2H, H-3), 2.51 (dd, *J* = 8.1, 6.6 Hz, 2H, H-2), 2.28 (s, 3H, H-11); ^13^C NMR (151 MHz, DMSO) *δ*: 170.46 (C-1), 157.93 (C-4), 139.50 (C-8), 139.36 (C-1′), 138.47 (C-2″), 132.34 (C-5), 129.60 (C-7, 9), 129.15 (C-3′, 5′), 128.79 (C-3″, 7″), 128.27 (C-4″,6″), 128.14 (C-5″), 126.56 (C-6, 10), 123.77 (C-4′), 119.72 (C-2′, 6′), 75.80 (C-1″), 33.26 (C-2), 22.52 (C-3), 21.21 (C-11); ESIMS: *m*/*z* 373.2 [M + H]^+^, 395.1 [M + Na]^+^, calc. for C_24_H_24_N_2_O_2_ (372.4596).

*(E)-4-(4-Methoxyphenyl)-4-[(methoxy)imino]-N-phenylbutanamide* (**5b-1**). Yield 78%, m.p. 125.6~126.5 °C; ^1^H NMR (600 MHz, DMSO) *δ*: 9.95 (s, 1H, H-NH), 7.68 (d, *J* = 8.6 Hz, 2H, H-6, 10), 7.61 (d, *J* = 8.0 Hz, 2H, H-2′, 6′), 7.32 (t, *J* = 7.7 Hz, 2H, H-3′, 5′), 7.06 (t, *J* = 7.3 Hz, 1H, H-4′), 7.00 (d, *J* = 8.6 Hz, 2H, H-7, 9), 3.94 (s, 3H, H-1″), 3.82 (s, 3H, H-11), 3.04–2.99 (m, 2H, H-3), 2.54 (t, *J* = 8.0 Hz, 2H, H-2); ^13^C NMR (151 MHz, DMSO) *δ*: 170.32 (C-1), 160.62 (C-8), 157.12 (C-4), 139.61 (C-1′), 129.12 (C-3′, 5′), 128.05 (C-6, 10), 127.57 (C-5), 123.54 (C-4′), 119.58 (C-2′, 6′), 114.39 (C-7, 9), 62.04 (C-1″), 55.67 (C-11), 33.45 (C-2), 22.38 (C-3); ESIMS: *m*/*z* 313.1551 [M + H]^+^, 355.1367 [M + Na]^+^, calc. for C_18_H_20_N_2_O_3_ (312.3630).

*(E)-4-(4-Methoxyphenyl)-4-[(benzyloxy)imino]-N-phenylbutanamide* (**5b-2**). Yield 79%, m.p. 119.0~119.6 °C; ^1^H NMR (600 MHz, DMSO) *δ*: 9.92 (s, 1H, H-NH), 7.66–7.60 (m, 2H, H-6, 10), 7.57 (d, *J* = 7.6 Hz, 2H, H-2′, 6′), 7.41 (d, *J* = 7.2 Hz, 2H, H-3″, 7″), 7.39–7.33 (m, 2H, H-4″, 6″), 7.32–7.26 (m, 3H, H-3′, 5′, 5″), 7.03 (t, *J* = 7.4 Hz, 1H, H-4′), 6.98–6.94 (m, 2H, H-7, 9), 5.19 (s, 2H, H-1″), 3.77 (s, 3H, H-11), 3.06–3.00 (m, 2H, H-3), 2.55–2.51 (m, 2H, H-2); ^13^C NMR (151 MHz, DMSO) *δ*: 170.30 (C-1), 160.66 (C-8), 157.55 (C-4), 139.59 (C-1′), 138.64 (C-2″), 129.13 (C-3′, 5′), 128.78 (C-3″, 7″), 128.31 (C-4″, 6″), 128.11 (C-6, 10), 128.10 (C-5″), 127.57 (C-5), 123.56 (C-4′), 119.58 (C-2′, 6′), 114.41 (C-7, 9), 75.74 (C-1″), 55.69 (C-11), 33.37 (C-2), 22.46 (C-3); ESIMS: *m*/*z* 389.1870 [M + H]^+^, 411.1676 [M + Na]^+^, calc. for C_24_H_24_N_2_O_3_ (388.4590).

*(E)-4-(4-Chlorophenyl)-4-[(methoxy)imino]-N-phenylbutanamide* (**5c-1**). Yield 76%, m.p. 151.3~152.4 °C; ^1^H NMR (600 MHz, DMSO) *δ*: 9.91 (s, 1H, H-NH), 7.71 (d, *J* = 8.5 Hz, 2H, H-6, 10), 7.55 (d, *J* = 7.6 Hz, 2H, H-2′, 6′), 7.48 (dd, *J* = 8.4, 1.4 Hz, 2H, H-7, 9), 7.28 (t, *J* = 7.8 Hz, 2H, H-3′, 5′), 7.03 (t, *J* = 7.3 Hz, 1H, H-4′), 3.93 (s, 3H, H-1″), 2.99 (t, *J* = 8.0 Hz, 2H, H-3), 2.54–2.47 (m, 2H, H-2); ^13^C NMR (151 MHz, DMSO) *δ*: 170.17 (C-1), 156.75 (C-4), 139.54 (C-1′), 134.46 (C-8), 134.13 (C-5), 129.13 (C-3′, 5′), 129.03 (C-7, 9), 128.47 (C-6, 10), 123.57 (C-4′), 119.57 (C-2′, 6′), 62.36 (C-1″), 33.21 (C-2), 22.35 (C-3); ESIMS: *m*/*z* 317.1048 [M + H]^+^, 339.0866 [M + Na]^+^, calc. for C_17_H_17_ClN_2_O_2_ (316.7821).

*(E)-4-(4-Chlorophenyl)-4-[(benzyloxy)imino]-N-phenylbutanamide* (**5c-1**). Yield 78%, m.p. 116.0~117.9 °C; ^1^H NMR (600 MHz, DMSO) *δ*: 9.95 (s, 1H, H-NH), 7.70 (t, *J* = 8.3 Hz, 2H, H-6, 10), 7.56 (dd, *J* = 22.3, 8.0 Hz, 2H, H-2′, 6′), 7.47 (d, *J* = 7.2 Hz, 2H, H-7, 9), 7.42 (t, *J* = 6.9 Hz, 2H, H-3″, 7″), 7.36 (dd, *J* = 6.4, 5.3 Hz, 2H, H-4″, 6″), 7.33–7.25 (m, 3H, H-3′, 5′, 5″), 7.03 (dd, *J* = 12.2, 6.3 Hz, 1H, H-4′), 5.22 (s, 2H, H-1″), 3.13–2.98 (m, 2H, H-3), 2.55 (dt, *J* = 16.4, 8.2 Hz, 2H, H-2); ^13^C NMR (151 MHz, DMSO) *δ*: 170.16 (C-1), 157.17 (C-4), 139.58 (C-1′), 138.36 (C-2″), 134.54 (C-8), 134.14 (C-5), 129.12 (C-3′, 5′), 129.04 (C-7, 9), 128.81 (C-3″, 7″), 128.50 (C-6, 10), 128.34 (C-4″, 6″), 128.19 (C-5″), 123.57 (C-4′), 119.59 (C-2′, 6′), 76.08 (C-1″), 33.18 (C-2), 22.46 (C-3); ESIMS: *m*/*z* 393.1370 [M + H]^+^, 415.1164 [M + Na]^+^, calc. for C_23_H_21_ClN_2_O_2_ (392.8780).

### 3.3. Bio-Evaluation Methods

#### 3.3.1. Cells and Cell Culture

HepG2.2.15 (clonal cells derived from human hepatoma cell line G2) cells were provided by the Chinese Academy of Medical Sciences (P.R. China) and maintained in minimal essential medium (MEM) supplemented with 10% fetal bovine serum and 380 μg/mL of G418, 50 ug/mL of kanamycin, and 0.03% l-glutamine at 37 °C in a 5% CO_2_ atmosphere with 100% humidity [26,33].

#### 3.3.2. Drug Treatment

HepG2.2.15 cells were seeded at a density of 1 × 10^5^ cells/ml (200 mL/well) in 96-well plates and maintained at 37 °C for 24 h prior to extract addition, followed by treatment with various concentrations of drugs. Lamivudine (**3TC**) was served as the positive control. Cells were treated with drug-containing fresh medium every 3 d for up to 9 d in a time-dependent experiment. Medium was taken at the 9th day of the treatment and stored at −20 °C until analysis. The IC_50_ and selected index (SI) of each compound were calculated, respectively.

#### 3.3.3. Cell Toxicity

Logarithmically growing cells were seeded in 96-well culture plates at a density of 1 × 10^5^ cells/mL (200 mL/well). They were cultured for 24 h and then treated with various concentrations of drugs. Optical density (OD) values were read at 450 nm after 9 days and the percentage of cell death was calculated; the cells were treated with drug-containing fresh medium every 3 d for up to 9 d. After drug treatment, the cytotoxicity was measured using the MTT assay [45,46].

#### 3.3.4. Method for HBsAg and HBeAg Inhibition Assays

The levels of HBV surface antigen (HBsAg) and HBV e antigen (HBeAg) in the supernatant of the HepG2.2.15 cell were simultaneously detected using enzyme-linked immunosorbent assay (ELISA) kits (Rongsheng Biotechnology Co. Ltd., Shanghai, China) according to the manufacturer’s instructions.The synthesized derivatives were expressed as the concentration of compound that achieved 50% inhibition (IC_50_) to the secretion of HBsAg and HBeAg. The selectivity index (SI) was determined as the ratio of CC_50_ to IC_50,_ which is a major pharmaceutical parameter of estimates possible for future clinical application.

## 4. Conclusions

In a summary, most of the compounds in the series of oxime ethers with a C_6_-C_4_ fragment, which were designed and virtually bioactively screened by docking with a target protein, showed anti-HBV activities in an in vitro assay. Among them, compound **5c-1** showed the most potent activity inhibiting HBsAg secretion (IC_50_ = 39.93 μM, SI = 28.51). The results of bioactive screening showed that the stronger the compounds bound to a target protein (human leukocyte antigen A protein) in docking, the more active they were for anti-HBV in vitro.

## Figures and Tables

**Table 1:** Docking results of synthesized compounds with 3OX8.

Compound	S-Score (kcal/mol)	Distance (Å)	Residue	Site of Actions
**3a-1**	−9.1113	2.72	Tyr27	N of -C=N-
**3a-2**	−9.7265	2.93	Tyr27	N of -C=N-
**3b-1**	−10.4709	2.94	Tyr26	N of -C=N-
**3b-2**	−11.2203	2.53	Tyr26	O of -C=O
		2.83	Tyr27	N of -C=N-
**3c-1**	−9.1497	3.09	Tyr63	N of -C=N-
**3c-2**	−10.0032	2.63	Tyr27	O of -C=O
		2.16	Tyr63	O of -C=O
**4a-1**	−9.9143	3.01	Tyr26	N of -C=N-
**4a-2**	−11.0692	2.94	Tyr63	O of -C=O
**4b-1**	−10.4782	2.82	Tyr26	N of -C=N-
**4b-2**	−10.8746	2.82	Tyr63	N of -C=N-
**4c-1**	−10.0261	3.01	Tyr27	N of -C=N-
**4c-2**	−10.3516	2.81	Tyr27	N of -C=N-
		2.73	Tyr63	N of -C=N-
**5a-1**	−9.9471	2.40	Tyr26	N of -C=N-
**5a-2**	−10.3332	1.96	Asp30	H of -N-H
		2.52	Tyr63	N of -C=N-
**5b-1**	−10.2883	2.75	Tyr63	N of -C=N-
**5b-2**	−12.0185	2.66	Tyr63	O of -C=O
**5c-1**	−9.9406	2.50	Tyr27	O of -C=O
		2.44	Tyr63	O of -C=O
**5c-2**	−11.2609	2.92	Tyr63	N of -C=N-

**Table 2:** Inhibition on the secretion of HBsAg, HBeAg and cytotoxicity of compounds in 9 days.

Compound	TC_50_^a^ (μM)	HBsAg ^b^		HBeAg ^c^	
		IC_50_^d^ (μM)	SI ^e^	IC_50_^d^ (μM)	SI ^e^
**3a-1**	>1500	292.73	>5.12	- ^f^	-
**3a-2**	>1500	-	-	-	-
**3b-1**	1383.74	-	-	-	-
**3b-2**	429.88	86.85	4.95	-	-
**3c-1**	450.67	224.82	2.00	-	-
**3c-2**	127.10	94.71	1.34	93.91	1.34
**4a-1**	>1500	154.50	>9.71	-	-
**4a-2**	>1500	-	-	-	-
**4b-1**	347.55	127.68	2.72	-	-
**4b-2**	1153.49	233.60	4.94	-	-
**4c-1**	>1500	-	-	-	-
**4c-2**	>1500	-	-	-	-
**5a-1**	>1500	74.92	>20.02	273.87	>5.48
**5a-2**	>1500	156.27	>9.60	220.09	>6.82
**5b-1**	473.25	207.63	2.28	-	-
**5b-2**	>1500	101.19	>14.82	191.58	>7.83
**5c-1**	1138.45	39.93	28.51	245.96	4.63
**5c-1**	>1500	-	-	-	-
**3TC ^g^**	517.40	290.73	1.78	358.59	1.44

^a^ TC_50_ is 50% cytotoxicity concentration in HepG 2.2. 15 cell; ^b^ HBsAg: hepatitis B surface antigen; ^c^ HBeAg: hepatitis B e antigen; ^d^ IC_50_ is 50% inhibitory concentration; ^e^ SI (selectivity index) = TC_50_/IC_50_; ^f^ The inhibition ratio less than 50% in the test concentration range; ^g^ Lamivudine (**3TC**) as the positive control.
